# Cannabinoid syndrome in cannabis dependence: a case report

**DOI:** 10.1192/j.eurpsy.2024.816

**Published:** 2024-08-27

**Authors:** C. Pichilingue Reto, A. M. Jose Rojo, M. B. Carrillo Reche

**Affiliations:** Psychiatry, Benito Menni CASM, Barcelona, Spain

## Abstract

**Introduction:**

A 36-year-old man with a history of cannabis use disorder since age 16, consuming 8-10 units/day, experienced irritability and tremors upon reducing consumption. His psychiatric issues emerged in 2020, marked by anxiety, abdominal pain, and severe vomiting, leading to a dyspepsia diagnosis. Subsequently, he received psychiatric care at CAS Hospitalet, diagnosed with severe cannabis use disorder. No prior inpatient admissions occurred.

**Objectives:**

Our project aims to show a case report and summarize the available evidence on cannabinoid hyperemesis syndrome (CHS).

**Methods:**

In May 2023, he voluntarily sought admission to Barcelona’s “Hospital Sant Pau,” aiming for cannabis detox and treatment of cannabinoid hyperemesis. He’d endured years of intense abdominal pain, nausea, and vomiting, worsening over the last two years, with uncontrollable vomiting hindering daily life. Admission saw reduced cannabis use to 3-4 units/day. Inpatient care revealed anticipatory anxiety, rumination, and somatic anxiety, accompanied by distal tremors and internal restlessness due to abdominal discomfort, partially alleviated by 5-10 mg of diazepam.

**Results:**

Treatment included domperidone 10mg/8h, haloperidol drops (5-10 drops/8h), capsaicin ointment, hot showers, and cryotherapy, resulting in gradual relief from abdominal pain. Moderate cravings for tobacco and cannabis led to acetylcysteine 600mg/12h and gabapentin up to 1200mg/8h. Gastric discomfort with SSRIs led to vortioxetine 10 mg/day, well-tolerated with a positive response. Consultation with the GI department confirmed the treatment’s efficacy, emphasizing cannabis abstinence. Upon discharge, cannabinoid hyperemesis symptoms markedly improved, and the patient was referred to “Hospital de Dia.”

**Conclusions:**

CHS is a cyclic vomiting syndrome, preceded by daily to weekly chronic longstanding use of cannabis that can be difficult to diagnose and treat(1,3,4). It is unique in presentation, because of the cannabis’s biphasic effect as anti-emetic at low doses and pro-emetic at higher doses, and the association with pathological hot water bathing (2). The major characteristics are as follows: history of regular cannabis for any duration of time (100%), cyclic nausea and vomiting (100%), resolution of symptoms after stopping cannabis (96.8%), compulsive hot baths with symptom relief (92.3%), male predominance (72.9%), abdominal pain (85.1%), and at least weekly cannabis use (97.4%)(1). Treatments such as topical capsaicin, haloperidol, benzodiazepines, and propranolol have shown symptom relief (3) whereas opioids should be avoided (4). Cannabis cessation appears to be the best treatment (1,3).

**References:**

1. Sorensen et al. *Journal of Medical Toxicology* 13 (2017):71-87.
2. Perisetti et al. *Annals of gastroenterology* 33.6 (2020):571.
3. Senderovich et al. *Medical Principles and Practice* 31.1 (2022):29-38.
4. Leu, et al. *Journal of Emergency Nursing* 47.3 (2021): 483-486

**Disclosure of Interest:**

None Declared

